# Comment on “Lessons from Toxicology: Developing a 21st-Century Paradigm for Medical Research”

**DOI:** 10.1289/ehp.1511148

**Published:** 2016-05-01

**Authors:** Ray Greek

**Affiliations:** Americans for Medical Advancement, Goleta, California, USA

The Brief Communication by Langley et al. was exceptional in its presentation of genomics and epigenomics, the discussion of the extrinsic and intrinsic causes of disease, and its outline of why a paradigm shift to human-based research is needed. I was disappointed however, to find that the authors relied on largely empirical evidence and observation and failed to include the very strong theoretical reasons for the failure of the current paradigm.

The theoretical grounding for the empirical evidence and observations cited by Langley et al. can be summarized as follows. A concept I developed called Trans-Species Modeling Theory (TSMT) states: “While trans-species extrapolation is possible when perturbations concern lower levels of organization or when studying morphology and function on the gross level, one evolved, complex system will not be of predictive value for another when the perturbation affects higher levels of organization” (Greek and Hansen 2013).

Humans and animals are examples of complex systems, which differ from simple systems in various important features. Complex systems exhibit a hierarchy of organization, with higher levels developing out of lower levels. Disease and drug responses occur at higher levels of organization and thus are difficult to model. Complex systems are highly dependent on initial conditions, demonstrate emergent properties, and are more than the sum of their parts. This limits the amount that we can learn about complex systems by using reductionism alone.

The initial conditions that concern biomedical research revolve around the genome of the patient. Genomes vary among individual humans and lead to important variation in drug response and disease susceptibility. Even monozygotic twins with their very minimal variation in genomes can respond differently to drugs and disease (Alexanderson and Borga 1972; Bell and Spector 2011; Czyz et al. 2012). The same has been demonstrated among ethnicities (Haiman et al. 2006) and between the sexes (Simon 2005). Even different strains of mice can respond dramatically differently to perturbations (Morange 2001, Belmaker et al. 2012). The striking divergence of responses to perturbations because of very small changes in initial conditions is illustrated by the divergence of weather outcomes demonstrated by Lorenz in his model of chaotic systems (Lorenz 1963) and has been reproduced in living systems (West 2006). Interspecies genome variation, including variation of the regulatory genome, is even more dramatic than the interindividual variations we are accustomed to observing (Romero et al. 2012).

Sir Arthur Eddington (2014) stated, “It is also a good rule not to put overmuch confidence in the observational results that are put forward until they have been confirmed by theory.” TSMT places the position of Langley et al. in the context of science in general and explains why animal models will continue to fail as predictive models for humans: Evolved, complex systems will continue to respond differently to perturbations like drugs and disease regardless of additions or deletions in the genome.
